# The impact of moderate endurance exercise on cardiac telomeres and cardiovascular remodeling in obese rats

**DOI:** 10.3389/fcvm.2022.1080077

**Published:** 2023-01-06

**Authors:** Maria Donatella Semeraro, Antonio Paolo Beltrami, Feras Kharrat, Gunter Almer, Simon Sedej, Wilfried Renner, Hans-Jürgen Gruber, Francesco Curcio, Markus Herrmann

**Affiliations:** ^1^Clinical Institute of Medical and Chemical Laboratory Diagnostics, Medical University of Graz, Graz, Austria; ^2^Department of Medicine (DAME), University of Udine, Udine, Italy; ^3^Department of Cardiology, Medical University of Graz, Graz, Austria; ^4^BTM BioTechMed-Graz, Graz, Austria; ^5^Faculty of Medicine, University of Maribor, Maribor, Slovenia

**Keywords:** cardiovascular remodeling, high-fat diet, moderate endurance exercise, cardiomyocyte senescence, telomere length, telomerase, shelterin, Sprague-Dawley rats

## Abstract

**Introduction:**

Hypercaloric nutrition and physical inactivity cause obesity, a potential driver of myocardial apoptosis and senescence that may accelerate cardiac aging. Although physical activity reduces mortality, its impact on myocardial aging is insufficiently understood. Here we investigated the effects of a hypercaloric high-fat diet (HFD) and regular exercise training on cardiac cells telomeres and histomorphometric indices of cardiac aging.

**Methods:**

Ninety-six 4-months old female Sprague-Dawley rats were fed for 10 months normal (ND) or a HFD diet. Half of the animals in each group performed 30 min treadmill-running sessions on 5 consecutive days per week. At study end, cardiomyocyte cross-sectional area (CSA), interstitial collagen content, vascular density, apoptotic and senescent cells, relative telomere length (RTL), and expression of telomerase-reverse transcriptase (*Tert*) as marker of telomere-related senescence and apoptosis were analyzed.

**Results:**

Compared to ND, the HFD group developed obesity, higher CSA, lower capillary density and tended to have more apoptotic cardiomyocytes and interstitials cells. Myocardial RTL and the expression of *Terf-1* and *Terf-2* were comparable in sedentary HFD and ND animals. In the HFD group, regular moderate endurance exercise improved myocardial vascularization, but had no effect on CSA or apoptosis. Notably, the combination of exercise and HFD increased senescence when compared to sedentary ND or HFD, and reduced RTL when compared to exercise ND animals. Exercising HFD animals also showed a trend toward higher *Tert* expression compared to all other groups. In addition, exercise reduced *Terf-1* expression regardless of diet.

**Conclusion:**

HFD-induced obesity showed no effects on myocardial telomeres and induced only mild morphologic alterations. Summarized, long-term moderate endurance exercise partially reverses HFD-induced effects but may even trigger cardiac remodeling in the context of obesity.

## 1. Introduction

Despite continuously improving diagnostic and therapeutic strategies, cardiovascular disease (CVD) still causes over 18.5 million deaths per year worldwide (1). The aging cardiovascular system is characterized by vascular changes, such as endothelial dysfunction, intimal thickening, and increased arterial stiffness. Ultimately, these age-related changes increase arterial pressure and impose mechanical stress on the heart. In response, the myocardium develops hypertrophy, leading to left ventricular (LV) diastolic, and eventually systolic, dysfunction (2–4). While cardiovascular aging is inevitable, lifestyle factors, such as diet and physical activity, have a major impact on this complex process. Although there is substantial evidence demonstrating adverse effects of unhealthy eating habits, obesity, and physical inactivity on the cardiovascular system, the underlying mechanisms are still insufficiently understood. Especially the interaction between dietary habits and physical activity is poorly studied. In Western societies, many people try to compensate unhealthy eating habits with exercise, but evidence for the efficacy of this approach is lacking.

A hypercaloric nutrition with high intake of saturated fat in combination with a sedentary lifestyle leads to obesity and metabolic syndrome (5). Both conditions are associated with increased mortality due to premature CVD (6), LV hypertrophy (7), and diastolic dysfunction, that, coupled with left atrial (LA) enlargement, is a risk factor for atrial fibrillation (8). In contrast, aerobic training has favorable effects on vascular and myocardial structure and function (9), which reduces CVD risk and cardiovascular mortality (10, 11). Large studies have consistently shown that 1 h per day of walking or 3 h per week of moderate-intensity jogging reduces mortality risk by up to 40% (10, 11). Regular exercise favors physiologic over pathologic hypertrophy (12), promotes myocardial autophagy (13), and ensures a healthy vascularization of the heart with adequate vascular density (14).

Recently, senescent cells have been identified as important promotors of cardiac aging and CVD (15). Cellular senescence refers to the reversible or irreversible withdrawal of cells from the cell cycle in response to a variety of stressors, such as telomere attrition, oncogene activation, genotoxic agents, mitochondrial dysfunction, inflammatory cytokines, and oxidative stress (16). *In vivo* experiments have shown that the removal of senescent cells prolongs the median life span of mice (17). These observations have sparked speculations that an unhealthy nutrition and a sedentary lifestyle promote cardiac aging and CVD through accelerated telomere shortening. Critically short telomeres are known to induce senescence and apoptosis (18). Physical activity is believed to preserve telomere length by increasing telomerase activity (19–22), an enzyme that counteracts telomere shortening through the addition of new nucleotides to telomeric ends. An upregulation of *Tert* expression in pre-senescent cells has been shown also to slow down senescence and tumorigenesis (23). Furthermore, exercise seems to modulate the expression of shelterin proteins, such as TRF2, which are essential for the protection of telomeres and their function (24).

Although the concept that lifestyle interventions may modulate telomere shortening and cardiac senescence by regulating telomerase activity and shelterin expression has attracted much attention, existing evidence is limited. Human studies that show differences in telomere biology between athletes and sedentary controls are mostly cross-sectional and focused on blood leucocytes rather than cells of the cardiovascular system (21, 25). So far, only one intervention study has shown a preservation of leucocyte telomere length and an induction of telomerase after 6 months of aerobic endurance exercise or high-intensity interval training, but not resistance training (21). Furthermore, results from the very few existing animal studies are also inconsistent. While voluntary wheel-running prevented cellular senescence in the thoracic aorta of C57/BL6 mice (25), it had no impact on the cardiac telomere length (26). In contrast, 1 year of voluntary wheel running attenuated the age-related shortening of telomeres in cardiomyocytes of CAST/EiJ mice (27).

Considering the lack of long-term studies that addressed the interaction between western-type diet and regular physical activity on telomere biology and cardiac aging, here we explored the impact of regular moderate exercise and a hypercaloric high-fat diet on cardiovascular remodeling, myocardial telomeres, senescence, and apoptosis.

## 2. Results

### 2.1. Impact of HFD and exercise on body weight and myocardial mass

Of the 96 rats that have been included, six had to be sacrificed before the end of the study because of general health issues. Additional 18 animals were excluded from statistical analyses because they developed tumors. Of note, animals on HFD were more frequently affected by tumors than animals on ND (36 vs. 4% respectively, *p* = 1.289 × 10^−4^). Interestingly, exercise did neither affect tumor incidence (coND vs. exeND, *p* = 0.975; coHFD vs. exeHFD, *p* = 0.347) nor the tumor types that were encountered upon HFD feeding (*p* = 0.197).

**Figure 1:**
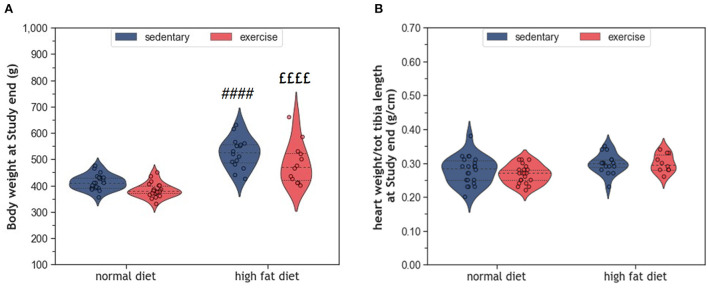
**(A)** Violin plots showing the distribution of body weight and **(B)** Normalized heart weight; the dashed line represents the group median whereas the dotted lines show the 25th−75th interquartile range. The circles indicate the scatter of the data. Group differences have been calculated by two-way ANOVA with Sidak *post-hoc* tests for multiple comparisons. *p-*values are only shown for pre-specified group comparisons. ^####^ *p* < 0.0001 compared to the sedentary normal diet control group; ^££££^ *p* < 0.0001 compared to the exercise normal diet group.

During the 10 months study period, sedentary ND rats (coND) showed an average increase in body weight corresponding to ~17% (*p* < 0.0001). At the same time, heart weight increased by 7% (*p* = 0.029), when adjusted for tibia length as an indicator of body size. Feeding the animals with HFD resulted in higher body weight at end of the study than in the respective ND controls (Figure 1A). In sedentary animals, body weight differed by 110 g (*p* < 0.0001), whereas their exercising counterparts showed a difference of 102 g (*p* < 0.0001). Although the impact of exercise on body weight was rather small, factorial ANOVA confirmed that both factors, diet (*F* = 83.38, *p* = 2.28 × 10^−13^) and exercise (*F* = 9.05, *p* = 0.004), had a significant influence on body weight. However, no significant interaction between diet and exercise was detected (*F* = 0.13, *p* = 0.72). In contrast to body weight, adjusted heart weight was only affected by HFD (*F* = 10.07, *p* = 0.002), but not exercise (Figure 1B). Again, there was no interaction between diet and exercise.

### 2.2. Impact of HFD and exercise on myocardial hypertrophy and fibrosis

**Figure 2:**
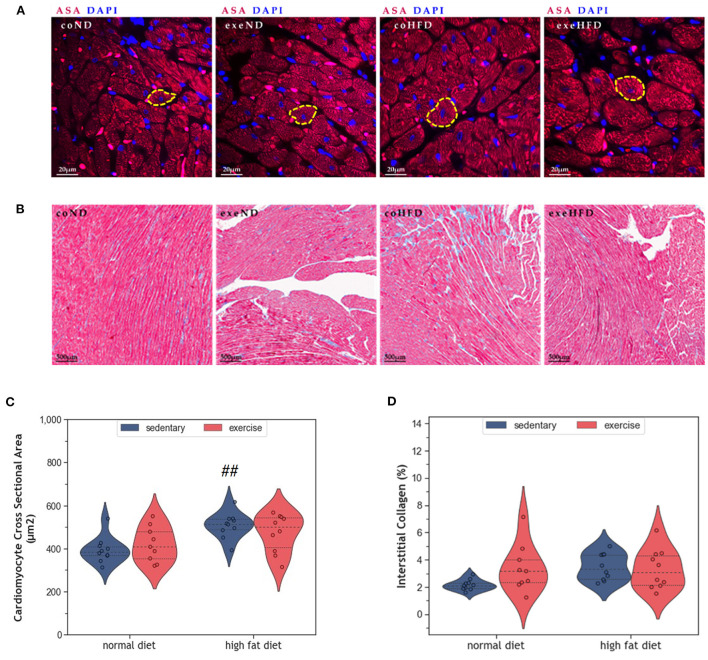
**(A)** Representative histological images of left ventricular cardiomyocytes showing a greater CSA in HFD animals. The sections have been stained with fluorescent anti -Sarcomeric alpha Actin (ASA) antibody and 4′,6-diamidino-2-phenylindole (DAPI). **(B)** Representative histological images of left ventricular myocardial tissue showing comparable amounts of interstitial collagen. The slides have been stained with Masson Trichrome and Haematoxylin-Eosin. **(C)** Violin plot showing the distribution of CSA in the different experimental groups. **(D)** Violin plot showing the distribution of interstitial collagen (%) in the different experimental groups. From each experimental group 10 animals were selected for histomorphometry analysis. Statistical differences between groups were analyzed by two-way ANOVA with Sidak *post-hoc* tests for multiple comparisons. *p* values are only shown for pre-specified group comparisons. ^##^*p* < 0.01 compared to the sedentary normal diet group.

Figure 2A provides representative histological images illustrating a greater cross-sectional area (CSA) in HFD-fed animals. Statistical comparison of CSA in coHFD and coND revealed significant hypertrophy of cardiomyocytes in obese animals (*p* = 0.008, Figure 2C). Factorial ANOVA confirmed HFD (*F* = 12.6, *p* = 0.001), but not exercise, as a significant modulator of cardiac hypertrophy. In contrast, HFD had no impact on interstitial collagen content (Figures 2B, D), an indicator of cardiac fibrosis.

### 2.3. Impact of HFD and exercise on myocardial apoptosis and senescence

**Figure 3:**
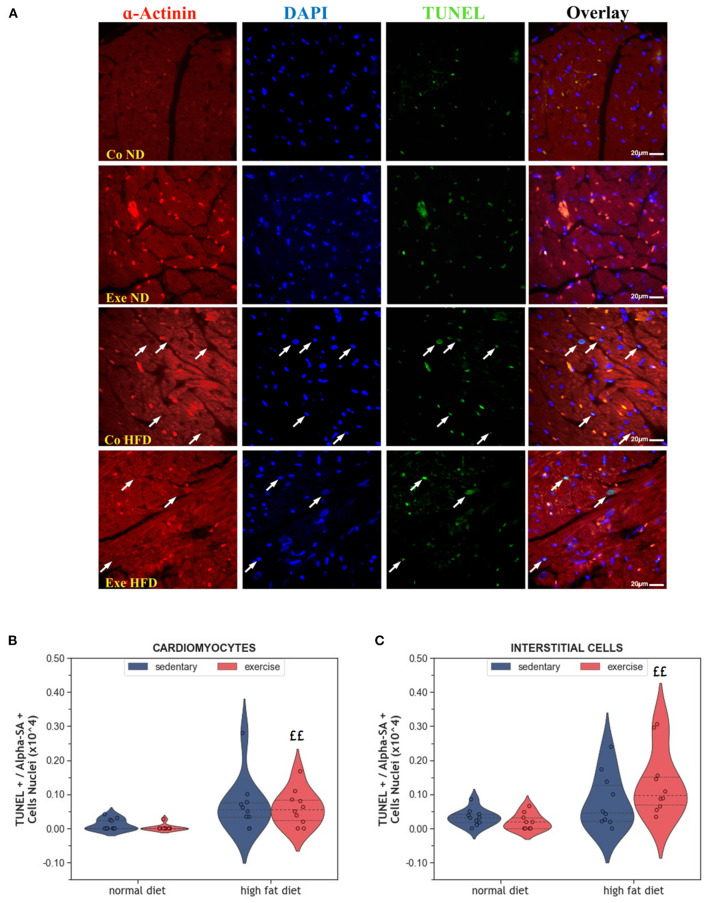
**(A)** Representative histological images of left ventricular sections showing an increased number of apoptotic cardiomyocytes in HFD groups. The sections have been stained with a commercial kit for terminal deoxynucleotidyl transferase dUTP nick end labeling (TUNEL) assay. Arrows point to apoptotic nuclei. **(B)** Violin plot showing the left ventricular fraction of Sarcomeric alpha-actinin positive cardiomyocytes that are also TUNEL positive. **(C)** Violin plot showing the left ventricular fraction of Sarcomeric alpha-actinin negative TUNEL positive interstitial cells. From each experimental group 10 animals were selected for histomorphometry analysis. Statistical differences between groups were calculated with the Kruskal-Wallis test adjusted by the Bonferroni correction for multiple tests. *p*-values are only shown for pre-specified group comparisons. ^££^*p* < 0.01 compared to the exercise normal diet group.

Apoptosis is a physiological process that eliminates old and damaged cells. The consumption of HFD increased the number of apoptotic cardiomyocytes, but this effect was only significant in exercising animals (*p* = 0.001, Figures 3A, B). Similar results were observed in interstitial cells of the myocardium (*p* = 0.001, Figure 3C).

**Figure 4:**
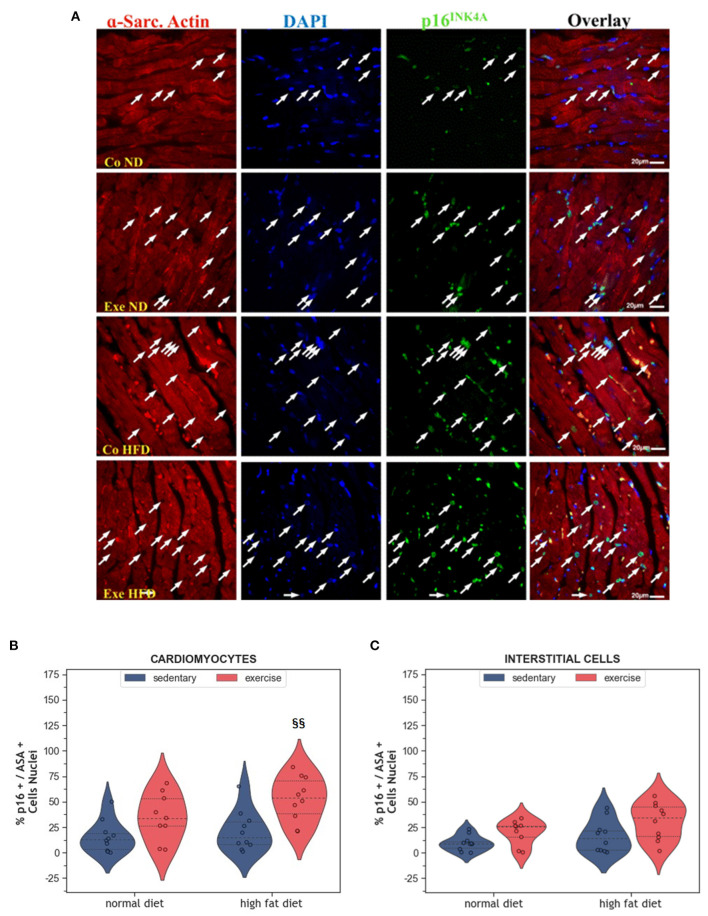
**(A)** Representative histological images of the left ventricle showing an increased number of senescent cardiomyocytes in HFD groups. The sections were co-immunostained with an anti-p16^INK4A^ antibody, an anti-Sarcomeric alpha Actin (ASA) antibody, and DAPI. Arrows point to senescent nuclei. **(B)** Violin plot showing the left ventricular fraction of Sarcomeric alpha-actin positive cardiomyocytes that are also p16^INK4A^ positive. **(C)** Violin plot showing the fraction of Sarcomeric alpha-actin negative p16^INK4A^ positive interstitial cells. From each experimental group 10 animals were selected for histomorphometry analysis. Statistical differences between groups were calculated with the two-way ANOVA with Sidak *post-hoc* tests for multiple comparisons. *p*-values are only shown for pre-specified group comparisons. ^§§^*p* < 0.01 compared to the high fat diet sedentary control group.

Senescence is another way of limiting the potential harm that can arise from old and damaged cells. Although senescent cells can no longer replicate, they remain metabolically active. They can be identified by immunohistochemistry using markers, such as p53, p21 or p16^INK4A^. Here we have analyzed p16^INK4A^, a final regulator of senescence that is mainly expressed when senescence is permanently established (28). In the present study, expression of the senescence marker p16^INK4A^ was not affected by HFD alone (Figures 4A–C). Instead, exercise increased the number of senescent cardiomyocytes in the myocardium (*p* = 0.008, Figures 4B, C). Factorial ANOVA confirmed the significant impact of exercise, but not HFD, on the number of senescent cardiomyocytes (*F* = 15.36, *p* < 0.001) and interstitial cells (*F* = 7.88, *p* = 0.008).

### 2.4. Impact of HFD and exercise on myocardial vascularization

**Figure 5:**
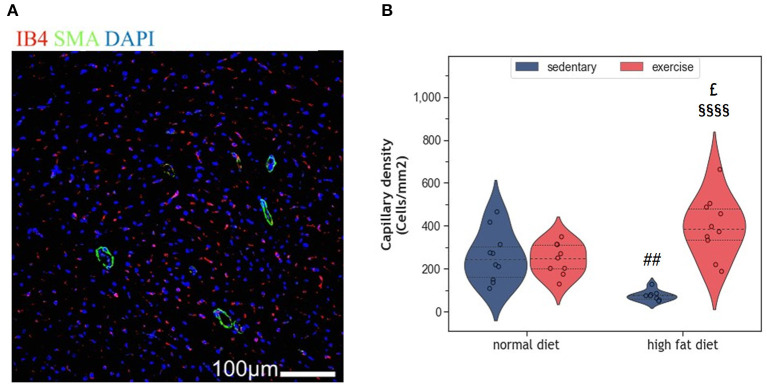
**(A)** Representative histological images of the left ventricle showing capillary density. The sections have been stained with anti-isolectin B4 (ILEB4), anti-Smooth Muscle alpha-Actin (SMA) antibodies and DAPI. **(B)** Violin plot showing the change in capillary density of the left ventricles of HFD groups. From each experimental group 10 animals were selected for histomorphometry analysis. Statistical differences between groups were calculated with the two-way ANOVA with Sidak *post-hoc* tests for multiple comparisons. *p*-values are only shown for pre-specified group comparisons. ^##^*p* < 0.01 compared to the sedentary normal diet group; ^£^*p* = 0.025 compared to the exercise normal diet group; ^§§§§^*p* < 0.00001 compared to the sedentary high fat diet group.

**Figure 6:**
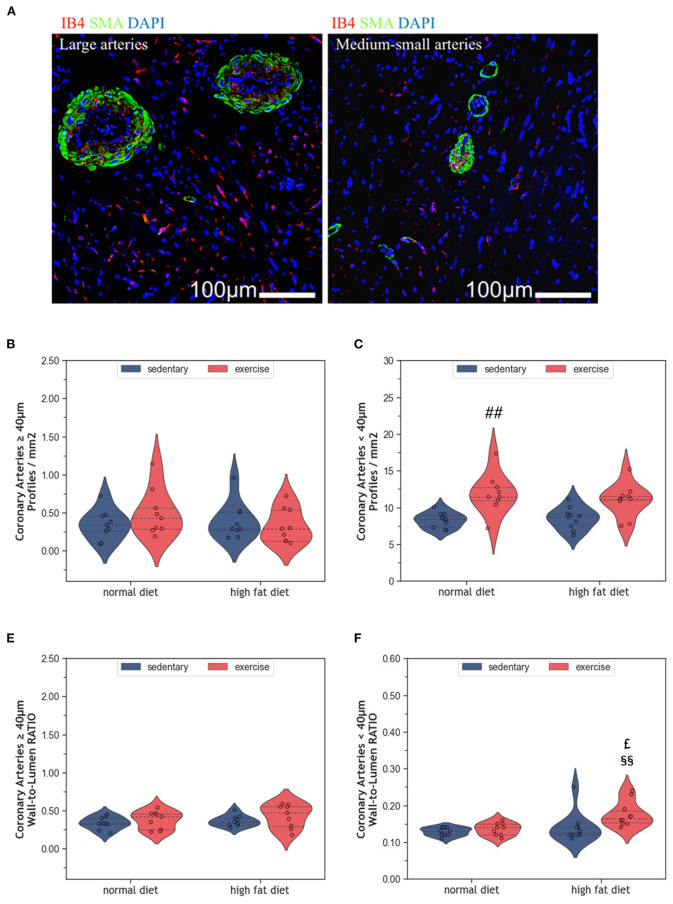
**(A)** Representative histological images of left ventricular myocardium showing coronary arteries of large and medium-small caliber. The sections were co-immunostained with ILEB4, anti-SMA antibody, and DAPI. **(B)** Violin plot showing the total number of large coronary arteries of left ventricular cardiomyocytes. **(C)** Violin plot showing the total number of medium-small coronary arteries of left ventricular cardiomyocytes. **(D)** Violin plot showing the wall-to-lumen ratio of large arteries. **(E)** Violin plot showing the wall-to-lumen ration of medium-small arteries. From each experimental group 10 animals were selected for histomorphometry analysis. Statistical differences between groups were calculated with the Kruskal-Wallis test adjusted by the Bonferroni correction for multiple tests. *p*-values are only shown for pre-specified group comparisons. ^##^*p* < 0.01 compared to the sedentary normal diet group; ^£^*p* = 0.039 compared to the exercise normal diet group; ^§§^*p* = 0.007 compared to the sedentary high fat diet group.

Myocardial vascularization is pivotal for tissue vitality and function. Histomorphometry analyses revealed a significant reduction of myocardial capillaries (*p* = 0.005, Figures 5A, B), but not arteries (Figures 6A–C), in HFD animals. In contrast, moderate regular exercise mitigated the reduction of myocardial capillaries in HFD animals (*p* < 0.05) but had no effect in ND animals (Figure 5B). The factorial ANOVA confirmed the previously described interaction of diet and exercise on capillary density (*F* = 24.04, *p* = 2.458 × 10^−5^). Also, exercising animals showed an increased number of small arteries with a diameter < 40 μm, but only upon ND, this effect reached statistical significance (*p* = 0.008, Figure 6C). In exeHFD animals, there was also a higher wall-to-lumen ratio, which was not observed in any of the other groups (*p* < 0.05, Figure 6E). Large myocardial arteries with a diameter equal to or >40 μm were neither affected by HFD nor by exercise (Figures 2B, D).

### 2.5. Impact of HFD and exercise on myocardial telomeres

**Figure 7:**
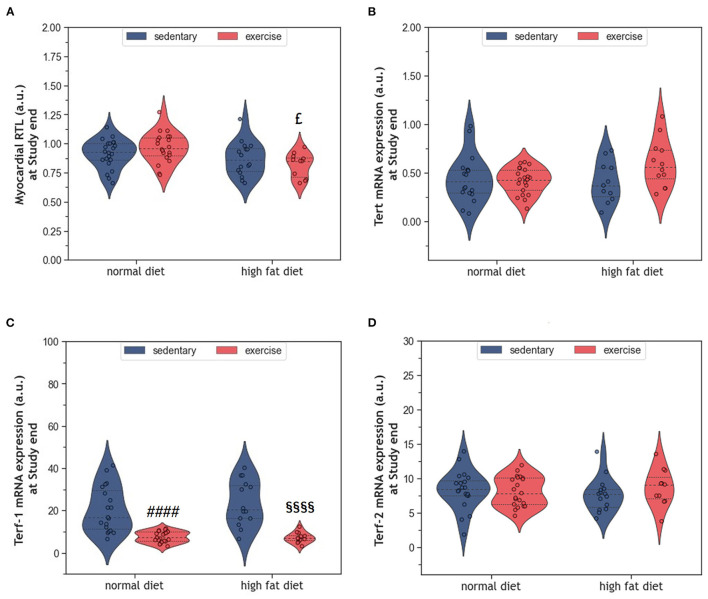
**(A)** Violin plot showing the myocardial RTL. **(B)** Violin plot showing the *Tert* mRNA gene expression. **(C, D)** Violin plot showing the *Terf-1* and *Terf-2* mRNA gene expression; the dashed line represents the group median whereas the dotted lines show the 25th−75th interquartile range. The circles indicate the scatter of the data. Group differences have been calculated in **(A–C)** by the two-way ANOVA with Sidak *post-hoc* tests for multiple comparisons; whereas in **(D)** with the Mann-Whitney *U*-test. ^£^*p* = 0.017 compared to the exercise normal diet control group; ^####^*p* < 0.0001 compared to the sedentary normal diet control group; ^§§§§^*p* < 0.0001 compared to the sedentary high fat diet group.

Telomere shortening is an established mechanism of aging that can trigger cell senescence or apoptosis when a critical length is reached. In the present study, HFD alone did not affect myocardial RTL (Figure 7A). Also, exercise alone did not modify myocardial RTL in exeND animals. Only exeHFD animals, where exercise and HFD were combined, showed slightly shorter telomeres than the respective controls. Factorial ANOVA confirmed the RTL shortening effect of HFD (*F* = 9.07, *p* = 0.004).

The expression of the catalytic unit of telomerase (*Tert*), an enzyme that can counteract telomere shortening by adding new hexanucleotides to telomeric ends, did not differ between groups (Figure 7B). However, factorial ANOVA showed a weak interaction of HFD and exercise, which was of borderline significance (*F* = 4.04, *p* = 0.049). Analyzing the mRNA expression of *Terf-1* and *Terf-2*, two proteins of the telomere-protecting shelterin complex, showed no significant effect of HFD (Figures 7C, D). In contrast, exercise strongly reduced the expression of *Terf-1*, but not *Terf-2* (Figures 7C, D). Factorial ANOVA confirmed a significant effect of exercise (*F* = 55.30, *p* = 3.77 × 10^−10^), but not HFD, on *Terf-1* expression.

## 3. Discussion

The long-term consumption of HFD induced obesity without affecting cardiac cells TL. In addition, there were only small alterations of the myocardium including moderate hypertrophy, a reduction of capillary density and a trend toward more apoptotic cardiomyocytes as well as interstitials cells. Moderate endurance exercise improved the HFD-induced reduction of myocardial vascularization but did not reverse hypertrophy or apoptosis. Furthermore, exercising obese animals had shorter telomeres than exercising lean animals. In lean animals, the exercise protocol had neither significant effect on myocardial RTL nor on various histological indices of aging.

Long-term HFD feeding did not affect cardiac telomeres and induced only minor structural changes in the myocardium. Such small effects may appear paradoxical when considering the broad spectrum of obesity-induced metabolic alterations in humans, such as dyslipidemia, hypertension, type 2 diabetes, and insulin resistance (8, 29). Obesity is further characterized by a hyperdynamic circulatory status with higher blood volume, increased heart rate, and an activated angiotensin-renin axis. The resulting hemodynamic stress triggers myocardial remodeling with myocardial hypertrophy and fibrosis. Ultimately, the progression of these metabolic and hemodynamic effects leads to cardiovascular disease, myocardial dysfunction, and heart failure (HF) (8, 29, 30). Some observational studies also suggest accelerated telomere attrition in the context of obesity and metabolic syndrome (31–33), whereas weight loss seems to preserve telomere length (34). However, existing studies in humans and rodents are inconsistent, and controversies remain (35–38). The modest cardiovascular alterations observed in the HFD animals of our study are most likely explained by the beef-tallow-based HFD that has a high content of saturated and polyunsaturated fat, but only moderate amounts of carbohydrates. In a previous study, this diet has been shown to provoke obesity with only small metabolic and inflammatory changes (39). This rather mild metabolic phenotype is probably due to species-specific differences in lipid trafficking and adipocyte lipid-storage capacity (40). The modest cardiovascular alterations despite marked obesity, support the concept that dyslipidemia and inflammation are crucial for the development and progression of coronary atherosclerosis, cardiac remodeling, and HF. In the absence of additional metabolic and inflammatory factors, an excess of dietary lipids may be of limited harm as they represent the predominant substrate for oxidative phosphorylation in cardiac mitochondria (41, 42). This concept is supported by human studies where isolated obesity was associated with a lower risk for CVD and cardiac dysfunction (43, 44). In the present study, HFD induced a significant increase in body weight (Figure 1A), but only mild LV hypertrophy without evidence of fibrosis (Figure 2). Moreover, myocardial hypertrophy is characterized by enlarged cardiomyocytes that push interstitial capillaries apart resulting in capillary rarefaction (45), which has also been observed in our HFD animals (Figure 5).

In contrast to the experimental model of our study, human obesity is usually the result of a diet that is high in saturated fat (SFA) and refined carbohydrates. Although a high dietary uptake of lipids with only a small fraction of polyunsaturated fatty acid (PUFA) species promotes inflammation and lipotoxicity (46, 47), excessive carbohydrate intake seems to dominate the detrimental cardiac effects in obese individuals (48–50). For example, hypertensive rats fed for 8 weeks a diet rich in carbohydrates showed an elevated end-systolic volume with a lower left ventricular ejection fraction, a higher number of apoptotic cardiomyocytes, and increased mortality when compared to rats on HFD (48). Also, nurturing men for several weeks with a high-sugar diet resulted in unfavorable changes in lipid metabolism and signs of hepatic lipid accumulation (51). In addition to metabolic, hemodynamic, and lipotoxic effects, also increased leptin concentrations promote myocardial hypertrophy and incident HF in obese individuals (52). For example, leptin treatment of cardiomyocytes from humans and neonatal rats has been found to induce TNF-α-/IL-6-mediated redox stress and cardiac hypertrophy (53–55). Despite high serum leptin, the HFD animals in the present study showed only a minor increase in oxidative-nitrosative stress (39), which may explain the lack of telomeric and apoptotic effects.

Regular exercise is believed to improve obesity, metabolic dysfunction, and chronic inflammation and slow down cellular aging, which ultimately reduces mortality. In a previous study we have shown that exercise ameliorated the pro-inflammatory profile of HFD-fed rats (39). While the present results indicate that exeHFD animals had also improved body weight and myocardial vascularization (Figures 1, 6), LV hypertrophy, interstitial fibrosis, and RTL were not altered (Figures 2, 7). Although there was no change in capillary density, the number of small arteries increased in response to exercise suggesting improved myocardial vascularization (Figure 6C). This concept is substantiated by an increase in RTL, *Terf-1*, and *Terf-2* expression in the aortic tissue of exercising animals (56). While moderate exercise has only mild cardiac effects, intensive exercise seems to induce cardiac hypertrophy, fibrosis, apoptosis, and inflammation in SD rats (57). Exercise-induced myocardial hypertrophy is a well-known physiologic reaction to high volume endurance exercise with a significant proportion of high-intensity training that is controlled by C/EBPbeta (12, 58). For example, 6-weeks of interval exercise with increasing intensity induced eccentric cardiac hypertrophy without fibrosis in 3-month-old male SD rats (59). In contrast, the metabolic stimulus of mild exercise, such as in the present study, is apparently insufficient to induce hypertrophy, but still has multiple beneficial anti-inflammatory, metabolic and vascular effects (58). However, a direct comparison of studies is often hampered by differences in age, sex, exercise protocol and nutrition.

Previous studies by Werner et al. demonstrated significant effects of voluntary wheel running on telomerase and shelterin expression as well as on survival pathways in blood leucocytes, aortic tissue, and the myocardium of 6-month-old C57/BI6 mice. Similar to the present study, these effects were not accompanied by alterations in myocardial RTL (25, 26). Others have shown an exercise-induced attenuation of myocardial telomere erosion in 1-year old CAST/EiJ mice (27). In this study, natural telomere shortening was accompanied by a decreased expression of the shelterins *Terf-1* and *Terf-2*. Therefore, contrasting results in existing studies may be due to differences in species, exercise protocols, study duration, and group size. While some studies worked with voluntary wheel running (25–27), others used motorized treadmills, where continuous running is imposed by mild electric stimuli (57). Although exercise volume and intensity are well standardized with this approach, the perceived psychological stress may be higher, which could explain some of the controversial effects. The fact that telomere shortening in cardiomyocytes is only detectable at older age may also be due to the post-mitotic nature of this cell type, where the end-replication problem does not exist. In such cells, telomere shortening is probably the result of other mechanisms that involve typical age-related effects, including metabolic dysfunction, chronic inflammation, and oxidative stress. Therefore, also the beneficial effects of lifestyle factors, such as exercise, may become more evident at older age. Notably, there is some evidence suggesting that the association between age and RTL is stronger in males than in females (60). Therefore, it cannot be excluded that age and lifestyle factors have more pronounced effects on the cardiovascular system in males than in females. However, this hypothesis is purely speculative, and the use of both sexes is warranted in future aging studies.

While all studies discussed before investigated the effects of exercise in lean animals, here we explored the effects of regular moderate running exercise in the context of obesity. Our exercise protocol reversed the reduction of myocardial vascularization in obese HFD animals (Figure 6) but did not affect the size of cardiomyocytes or myocardial apoptosis (Figures 2–4). Despite the improvement of myocardial vascularization and the lack of fibrotic tissue, exercise was accompanied by evidence of arterial remodeling (Figure 6E) which is one of the earliest signs of target organ damage in hypertension (61). Together with shorter telomeres (Figure 7A) and higher numbers of p16^INK4A^ positive cardiomyocytes (Figure 4B), the higher wall-to-lumen ratio in HFD animals questions the beneficial nature of forced endurance exercise in obese individuals. In analogy to observations by Agrimi et al., the psychological stress induced by frequent forced running might have caused an adaptive stress response with increased myocardial apoptosis, senescence, and oxidative-nitrosative stress (39, 62). Increased oxidative-nitrosative stress might be a potential mechanism that mediates adaptive stress responses in our animals including telomeric dysfunction. This hypothesis is supported by a positive correlation between the serum NOx concentration and cardiac *Terf-1* expression in our animals. A relationship between eNOS and telomere function in exercising mice has also been reported by Werner et al. (25). However, the exeHFD animals also showed a moderate upregulation of *Tert* expression (Figure 7B), which might reflect an altered DNA-damage-repair and checkpoint response to short-telomere-induced DNA damage (23). Thus, the upregulation of *Tert* expression in these animals could reflect a protective mechanism aiming to prevent myocardial degeneration due to premature telomere attrition and chronic cellular senescence.

In conclusion, isolated obesity induced by a hypercaloric HFD with low carbohydrate content has no telomeric effects in the myocardium and induces only mild morphologic alterations suggesting that this treatment does not significantly accelerate cardiac aging. Also, long-term moderate endurance exercise was not associated with clear cardioprotective effects. Therefore, obesity without additional risk factors or comorbidities may be less harmful than expected. Furthermore, moderate exercise appears to be of limited relevance for myocardial telomere biology and cardiovascular remodeling. Of note, the results from this study cannot easily be translated to humans where cardiovascular aging is usually a multifactorial process. In conjunction with the existing literature, our findings emphasize once more the importance of the experimental setting, which may profoundly impact the results.

## 4. Materials and methods

### 4.1. Animal and experimental protocol

Four-month-old healthy female SD rats (*n* = 96) with an average body weight of ~300 g were purchased from Janvier Labs (Le Genest-Saint-Isle, France) and kept in groups of three animals per cage under constant housing conditions on a 12 h light and 12 h dark cycle in the Core Facility Experimental Biomodels, Division of Biomedical Research of the Medical University of Graz (Austria). After 1 week of acclimatization, the animals were randomly assigned to receive a standard diet (ND) (Altromin, Lage, Germany) with 3,226 kcal/kg and 11% fat or a purified beef-tallow high-fat diet (HFD), rich in SFAs and MUFAs, in particular C16:0 (8.27%), C18:0 (6.06%), and C18:1 (12.29%), with 5,150 kcal/kg and 60% fat (ssniff, Soest, Germany). Food and tap water were provided *ad libitum*.

#### 4.1.1. Study design

Animals were randomly allocated to 4 groups, each consisting of 24 animals. After the acclimatization period, the animals were divided in a 1:1 ratio to receive either ND or HFD for the entire 10-month study period. Half of the ND and HFD-fed rats were assigned to a standardized exercise program that consisted of 30 min of running on a 5-lane treadmill (Panlab, Barcelona, Spain) on five consecutive days per week (in a time frame between 10:00 am and 2:00 pm) with 2 days of rest. Running speed was kept constant at 30 cm/s. These two groups are referred to as exeND and exeHFD. The remaining animals were used as sedentary controls and are referred to as coND and coHFD. Of note, only female SD rats were used in this study because the expected final weight upon HFD feeding was rather high (>500 g) and, considering that male SD rats naturally exceed the weight of females already on a normal diet chow, there was a risk that they would not fit into the appropriate space in our multilane treadmill. Furthermore, females are less aggressive than males, making them more suitable for long-term studies. After the exclusion of dropouts, 72 eligible animals were included in the final statistical analyses.

#### 4.1.2. Euthanasia and sample preparation

At the end of the 10-months study protocol, blood was drawn by heart puncture under deep isoflurane anesthesia (Forane, Abbott, Austria). Blood and plasma were collected using S-Monovette Serum-Gel tubes and S-Monovette Plasma-EDTA tubes (Sarstedt, Nümbrecht, Germany), respectively. Blood collection was performed in a non-fasting state. From 10 animals per group, the base and mid-ventricular portion of the myocardium were formalin-fixed for 24 h and stored in EtOH at 4°C until analysis. The samples have been rehydrated overnight in PBS at 4°C before sectioning. The apical portion of the ventricles was snap frozen from all animals and stored at −80°C for subsequent molecular analyses.

### 4.2. Analysis of relative telomere length in cardiac tissue

Approximately 10 mg derived from ventricular tissue were homogenized in 300μl Magna Pure Lysis Buffer (Roche, Vienna, Austria) using the MagnaLyser (Roche, Vienna, Austria). DNA was isolated with the MagNA Pure LC instrument (Roche, Vienna, Austria) using the Total Nucleic Isolation Kit (Roche, Vienna, Austria). Subsequently, relative telomere length (RTL) of peripheral blood mononuclear cells (PBMCs) was measured by quantitative real-time PCR (qPCR) using a protocol developed by Cawthon (63). This assay quantifies the ratio of average TL (T) to glyceraldehyde-3-phosphate dehydrogenase (GAPDH) as a single-copy reference gene (S). The single-copy gene is used as an amplification control for each sample and to determine the number of genome copies per sample. All qPCR analyses were performed on Thermocycler CFX384 TouchTM (Biorad, Feldkirchen, Germany) using the following primers:

- Telomere Forward: 5′-CGGTTTGTTTGGGTTTGGGTTTGGGTTTGGGTTTGGGTT-3′;
- Telomere Reverse: 3′-GGCTTGCCTTACCCTTACCCTTACCCTTACCCTTACCCT-5′;
- GAPDH Forward: 5′-CACCTAGACAAGGATGCAGAG-3′;
- GAPDH Reverse: 3′-GCATGACTGGAGGAATCACA-5′.

All primers have been purchased from Eurofins Genomics, Austria. Each run included a standard curve made by dilutions of isolated and pooled rat DNA from 21 different blood samples, to determine the quantity of the targeted templates. RTL has been calculated as the ratio of telomere quantity to single-copy reference gene quantity (T/S ratio).

### 4.3. The mRNA expression analyses in cardiac tissue

*Tert, Terf-1*, and *Terf-2* gene expression was analyzed in RNA extracts of ventricular tissue. 10 mg of tissue were homogenized in 300 μl Magna Pure Lysis Buffer (Roche, Vienna, Austria) using the MagnaLyser (Roche, Vienna, Austria). RNA was extracted from these homogenates with the Total Nucleic Isolation Kit (Roche, Vienna, Austria) on a MagNA Pure LC instrument (Roche, Vienna, Austria). Subsequently, the mRNA in these extracts was transcribed into cDNA using the QuantiTect Reverse Transcription kit (Qiagen, Hilden, Germany). Finally, mRNA expression of TERT, TERF-1, and TERF-2 was analyzed by qPCR with TaqMan probes (Life Technologies dba Invitrogen, Waltham, MA, USA). The expression of each target gene was calculated with the ΔΔCT method using β-actin as a reference gene. The sequences of the probes used are listed below:

- B-actin: 5′-CTTCCTTCCTGGGTATGGAATCCTG-3′;
- Tert: 5′-ATCGAGCAGAGCATCTCCATGAATG-3′;
- Terf-1: 5′-AAAACAGACATGGCTTTGGGAAGAA-3′;
- Terf-2: 5′-GAGAAAATTTAGACTGTTCCTTTGA-3′.

### 4.4. Histological analyses

The base and mid-ventricular portion of the myocardium was formalin-fixed. Following fixation, the pathologist (APB) subsampled rat hearts. The most proximal section of the base of the heart and the most distal transverse section of the heart (mid-section to apex junction) were included in the same paraffin-embedded (FFPE) block facing the section plan. 5 μm-thick sections were obtained from FFPE blocks, so that 2 plans of the same heart were included in each slide: base and mid-section. Then the slides were deparaffinized with xylene, rehydrated with four consecutive ethanol washes (100, 95, 80, and 70%) and a 3 min wash in distilled water, and underwent antigen retrieval in Sodium Citrate 0.1 M, pH = 6, for 40 min at 98°C. After a cool-down phase of at least 40 min, and three washing steps, one in distilled water (5 min) and two in 1X PBS (5 min each), deparaffinized tissue sections were stained. TUNEL staining was used to detect myocardial apoptosis. Apoptotic cardiac cells were detected after staining with anti-Sarcomeric alpha Actinin antibody. Percent apoptotic cardiac cells and cardiomyocytes (% ×10^4^) were calculated *via* the number of TUNEL-positive nuclei over the total number of cardiac cells or cardiomyocyte nuclei per field (at a magnification of 100×), respectively (64). Cardiomyocytes and non-cardiomyocytes senescence were detected with staining for p16^INK4A^ antibody and anti-alpha-SA antibody. The percentage of p16^INK4A^ expression was calculated from the p16^INK4A^ positive cardiomyocytes/non-cardiomyocytes normalized to the total number of cardiomyocytes per area.

Additionally, interstitial collagen was determined *via* Masson Trichrome and H&E-stained sections (HT15 Trichrome Stain kit; Sigma). To assess cardiomyocyte hypertrophy, we employed the cross-sectional area–CSA, a surrogate parameter (65). To evaluate artery morphology, we counted the number of vessels positive for Smooth Muscle Actin antibody on an entire transverse section of the upper myocardium and measured both their thickness and relative caliber. Artery dimensions and cardiomyocyte cross-sectional areas were computed employing ImageJ software (66). Vessel morphology was evaluated on 15 images of Isolectin B4 stained sections of the myocardium, taken at 400× magnification, employing the AngioTool free software (67).

### 4.5. Statistical analysis

Data are expressed as mean ± standard deviation. Gaussian distribution of the parameters was assessed employing the Kolmogorov-Smirnov and the Shapiro-Wilk test. Qualitative variables such as tumor frequency and diversity were assessed with Fisher's exact test or the Chi-squared test. Two-way ANOVA followed by Sidak *post-hoc* test was employed to dissect the impact that both diet and exercise exert on the observed parameters. Differences among more than two groups non-normally distributed were assayed with the Kruskal-Wallis test adjusted by the Bonferroni correction for multiple tests. IBM^®^ SPSS^®^ Statistics, version 26.0 for Windows, was used for explorative data analysis, and the level of acceptance of the null hypothesis was set at *p* < 0.05. Python programming language with Jupyter Notebook within the data science package Anaconda3 for Windows was used for data visualization.

## Data availability statement

The original contributions presented in the study are included in the article/supplementary material, further inquiries can be directed to the corresponding author.

## Ethics statement

The animal study was reviewed and approved by the responsible National Ethics Committee (GZ: 66.010/0070-V/3b/2018) and conducted in accordance with the guidelines of the Animal Care and Use Committee of the Ministry of Science and Research, Vienna, Austria.

## Author contributions

Conceptualization: MH, H-JG, and GA. Methodology and validation: AB, WR, and H-JG. Software, writing—original draft preparation, and visualization: MS. Formal analysis: H-JG, AB, and MS. Investigation: MS, AB, H-JG, GA, MH, and FK. Resources: FC and MH. Data curation: MS and AB. Writing—review and editing: AB, GA, SS, WR, H-JG, FC, and MH. Supervision: MH and AB. Project administration and funding acquisition: MH. All authors have read and agreed to the published version of the manuscript.
